# Successful Localization of the Source of Hemorrhage in Patient with Post-Whipple Surgery by ^99m^Tc-Labelled Red Blood Cell Scintigraphy

**DOI:** 10.1155/2018/1381203

**Published:** 2018-08-14

**Authors:** Ahmed Fathala, Alaa Alduraibi, Moheieldin M. Abouzied

**Affiliations:** Department of Radiology, Division Nuclear Medicine Division & Cardiothoracic Radiology Division, King Faisal Specialist Hospital & Research Centre, P.O. Box 3354, MBC 28, Riyadh 11211, Saudi Arabia

## Abstract

Gastrointestinal Bleeding Scintigraphy (GIBS) of ^99m^Tc-labelled red blood cells is a relatively simple examination to perform, with high diagnostic accuracy and a relatively lower radiation dose. A positive scan can either suggest surgery without further investigation or can indicate angiography, a more targeted procedure. Whipple pancreatoduodenectomy is most often performed for tumors of the head of the pancreas. Pancreatoduodenectomy has 30%–40% morbidity and mortality, and while post-pancreatoduodenectomy hemorrhage is seen in less than 10% of patients, it accounts for 11%–38% mortality. The role of imaging in patients to detect relative hemodynamic stability is essential. Computed tomography angiography (CTA) shows the cause, site, and nature of bleeding, while digital subtraction angiography (DSA) has a diagnostic as well as a therapeutic role. We present a patient who presented with active gastrointestinal bleeding (GI) bleeding after undergoing a Whipple procedure, to highlight the role of GIBS in the successful localization of a bleeding site and the guidance of digital DSA in the embolization and control of the active bleeding.

## 1. Introduction

Gastrointestinal Bleeding Scintigraphy (GIBS) of ^99m^Tc-labelled red blood cells blood cells is a relatively simple examination, where the scan is useful for diagnosing active gastrointestinal bleeding (GI bleeding) and for guiding therapeutic angiography. The technique is very sensitive and can detect bleeding rates of 0.05– 0.1 cc/min, while conventional angiography allows the detection of bleeding at rates of only 0.5–1.0 mi/min and above [1], and computed tomography angiography (CTA) can detect bleeding rates of 0.3–1 ml [2]. A positive scan can either indicate surgery without further investigation or can suggest a more targeted angiography procedure. Whipple pancreatoduodenectomy is most often performed for tumors of the head of the pancreas. Pancreatoduodenectomy has 30%–40% morbidity and mortality, while post-pancreatoduodenectomy hemorrhage accounts for 11%–38% mortality even though it is seen less than 10% of patients [3]. Several vascular lesions and sources have been discerned using this method. But it is essential to use imaging in patients to detect relative hemodynamic stability; CTA shows the cause, site, and nature of bleeding [4], while digital subtraction angiography (DSA) has a diagnostic as well as a therapeutic role [5].

There has been very little emphasis on the role and utilization of GIBS with a ^99^Tc-labelled red blood cell scan in the following clinical situation. We present a patient who presented with active GI bleeding after undergoing a Whipple procedure in order to highlight the role of GIBS in the successful localization of the bleeding site and the guidance of digital DSA in the embolization and control of the active bleeding.

## 2. Case Presentation

A 53-year-old patient was recently diagnosed with pancreatic cancer with obstructive jaundice, for which he underwent a Whipple procedure. Unfortunately, the procedure was complicated with a pancreatojejunostomy anastomosis leak, deep vein thrombosis deep vein thrombosis (DVT), and postoperative-bleeding. He was taken for exploratory laparotomy and a revision of gastrojejunostomy anastomosis without the successful localization of the source of the bleeding; later the same day, the patient underwent diagnostic DSA, which also failed to localize the source of the bleeding. GIBS was requested for better localization of the GI bleeding source.

**Figure 1:**
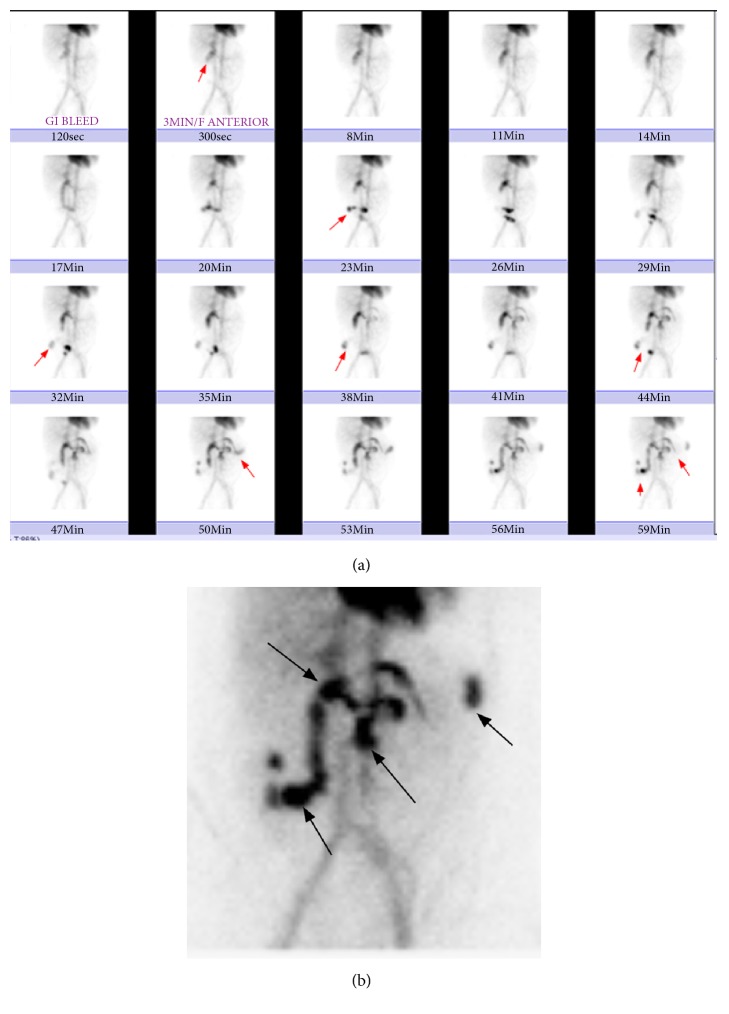
(a) Dynamic imaging sequence of a ^99^Tc-labelled erythrocyte scan. The initial image (2 min image) demonstrates normal biodistribution of the radiotracer. A focus of increasing intensity is noted in the right upper abdomen at the 3 min image (red arrow). This focus of abnormal accumulation of the radiotracer shows antegrade and retrograde movement, confirming the bowel lumen appearance, and it crosses the midline several times (on cine images). This pattern is compatible with small bowel bleeding. (b) This selected late image from the ^99^Tc-labelled erythrocyte bleeding scan (59 min image) shows the typical pattern of small bowel bleeding (multiple red arrows).

**Figure 2:**
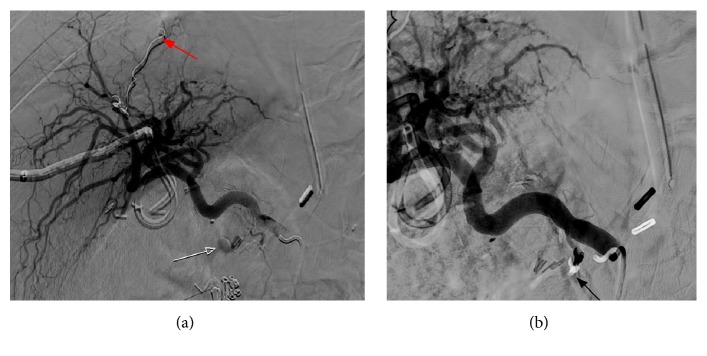
(a) Digital subtraction angiogram (DSA) with selective injection through the common hepatic artery shows active extravasation from a small artery arising from the gastroduodenal artery stump. The red arrow indicates previous coil embolization in the right hepatic artery branch, due to previous hepatic trauma from the prior insertion of a percutaneous transhepatic biliary drain. (b) DSA shows the embolization coil placed in the gastroduodenal artery stump with excellent hemostasis (arrow).

The study was positive for active bleeding, which had started primarily high in the right upper abdomen, supposedly from the region of the hepaticojejunostomy (Figures 1(a) and 1(b)). Therefore, the patient was transferred to the angiography suite for another diagnostic and therapeutic DSA. Selective catheterization of the superior mesenteric artery was performed followed by an angiogram, which showed no active contrast extravasation and identified no abnormality. Then selective catheterization of the celiac trunk was followed by an angiogram, which showed small contrast extravasation originating from the proximal common hepatic artery, most likely from the gastroduodenal artery stump (Figure 2(a)). Using a coaxial microcatheter/microwire utilizing an Echelon catheter and synchro-wire, contrast was injected for the selective catheterization of the small arterial branches originating from the proximal main common hepatic artery. A small extravasation was confirmed, and while the catheter remained in the same position, coil embolization was performed utilizing three coils measuring 2 mm. After that, an angiogram was performed that showed no extravasation and no abnormality (Figure 2(b)). And upper abdominal angiogram was also performed, again demonstrating no abnormalities. No immediate complications were encountered.

## 3. Discussion

GIBS contributed very significantly to the management of this postoperative high-risk patient; this procedure accurately confirmed that the patient was actively bleeding, it found the location of the bleed, and it provided guidance for radiologic intervention. The main advantage of GIBS compared to CTA is its ability to detect GI bleeding at a very low threshold, at a rate of 0.05–0.2ml/min [6]. The sensitivity and specificity of GIBS have been reported to be 93% and 95%, respectively [7, 8]. There are certain predictors of GIBS that correlate with the positivity of DSA, its prompt performance, and the higher intensity of the radiotracer accumulation at the site of the bleeding [9]. GIBS may be considered the initial test in the management of a post-Whipple patient presenting with hemorrhage, as it provides greater diagnostic accuracy, is relatively simple to perform, and uses a lower radiation dose than those of other methods.

An alternative diagnostic test for lower GI bleeding in post-Whipple patients is CTA, which is widely available, provides a faster diagnosis, particularly in patients who cannot be tested in other ways, and delineates other possible etiologies, such as vascular anomalies. But the drawbacks of CTA include higher relative costs, a higher radiation dose, limited time for evaluation, lower sensitivity compared to GIBS, and iodine contrast IV administration. GIBS is preferred, especially in the pediatric population, because of its lower radiation dose compared to CTA [10].

Several previous studies have suggested that GIBS can be effective as the sole tool in surgical planning [11], but others have suggested that it is useful only as a screening tool before angiography and intervention [12]. The value of SPECT/CT GIBS for localization, sensitivity, and specificity has yet to be investigated. Bleeding in the small bowel may be more difficult to detect and localize compared to colonic bleeding, since the location of the small bowel is more variable and more central, and it is more prone to overlap with vascular structures [13].

Post-pancreatoduodenectomy hemorrhage is seen in less than 10% of patients, but as mentioned, it accounts for 11%–38% of mortality [3]. Intramural hemorrhage may present as hematemesis, bleeding from the nasogastric tube, or melena. The possible sources of bleeding in post-pancreatoduodenectomy include the gastroduodenal artery stump, common and proper hepatic artery erosion, or splenic and inferior pancreaticoduodenal artery erosions or aneurysm, besides other etiologies [14].

## 4. Conclusion

Post-Whipple pancreatoduodenectomy hemorrhage is a common but important complication with high mortality. GIBS may be viewed as an initial diagnostic test in stable patients; it is simple and easy to perform, with the highest diagnostic accuracy and a relatively lower radiation dose, and it may guide therapeutic percutaneous radiologic intervention. CTA is an alternative diagnostic test resulting in faster diagnosis, it delineates other possible causes of bleeding such as vascular anomalies, and it can guide endovascular therapy. But CTA is more expensive, it has a higher radiation dose, and it needs iodinated contrast IV administration. DSA has a role in both diagnosis and intervention, but the role of DSA has changed from diagnostic to therapeutic. Radiologic intervention is preferred in the management of post-Whipple pancreatoduodenectomy hemorrhage, because of the lower resulting mortality.

## Data Availability

The case report data used to support the findings of this study are available from the corresponding author upon request.
